# Supplementary material for: Prevalence of anemia and its associated factors among adolescent girls on Weekly Iron Folic Acid supplementation (WIFAS) implemented and non-implemented schools at Tokha municipality, Kathmandu
Source: PLOS Glob Public Health. 2024 Jan 26;4(1):e0002515. doi: 10.1371/journal.pgph.0002515 (PMC10817172; doi:10.1371/journal.pgph.0002515)
Supplement: S1 File — (DOCX) [file pgph.0002515.s001.docx]

**S1 File: Self- administered questionnaire**

**Tribhuvan University**

**Institute of Medicine**

**Central Department of Public Health**

**Master in Public Health Nutrition 2021**

**Prevalence of anemia and its associated factors among adolescent girls on Weekly Iron Folic Acid supplementation (WIFAS) implemented and non-implemented schools at Tokha municipality, Kathmandu**

Name of the school……………………………...............................................................................

Type of school(Government/private) ……………...……………………………....………………

Address of the school………………………………………………...……Form No.….…………

Date of interview (DD/MM/YY) ……………………………

Please Tick (√) the options to answer the question.

| **SECTION A: SOCIO-DEMOGRAPHIC CHARACTERISTICS** | | | |
| --- | --- | --- | --- |
| S.N | Questions | Options | Code |
| 1. | Age in years (completed age) |  |  |
| 2. | Class |  |  |
| 3 | What is your first age of mensuration? |  |  |
| 4. | Marital status of respondents | Never married  Married/living together  Divorced/Separated  Widowed |  |
| 5. | Ethnicity | Bhramin/Chhetri  Dalit  Janajati  Madhesi  Muslim  Others (specify) |  |
| 6. | Religion | Hindu  Buddhist  Muslim  Kirat  Christian  Others (specify) |  |
| 7. | Type of Family | Nuclear  Joint/Extended |  |
| 8. | Educational status of Mother | **No education**  **Basic education (1-8)**  lower basic education (1-5)  upper basic education (6-8)  **Secondary (9-12)**  lower secondary (9-10)  upper secondary (11-12)  **More than secondary (13 and above)** |  |
| 9. | Educational status of father | **No education**  **Basic education (1-8)**  lower basic education (1-5)  upper basic education (6-8)  **Secondary (9-12)**  lower secondary (9-10)  upper secondary (11-12)  **More than secondary (13 and above)** |  |
| 10. | Father’s occupation | Agriculture  Housework  Government Service  Private business  Foreign Employment  Daily wages  Other (specify) |  |
| 11. | Mother’s occupation | Agriculture  Housework  Government Service  Private business  Foreign Employment  Daily wages  Other (specify) |  |

| **SECTION B: Knowledge on Anemia and Weekly iron folic acid supplementation (WIFAS)**  **Knowledge on Anemia** | | | |
| --- | --- | --- | --- |
| 12. | What does it mean to have anemia? | Not having enough blood  Having more blood  Running diarrhea  Don’t know |  |
| 13. | Do you know what causes anemia?  **Tick all that apply** | Iron and folic acid deficiency  Bacteria  Vitamin A and B12 Deficiency  Parasitic infection  having too much sugar  Blood loss due to menstruation  Don’t know |  |
| 14. | What are the symptoms of anemia?  **Tick all that apply** | Fatigue  Bleeding  Fast breathing  Cold  Paleness of skin  Chest pain  Fainting  Dizziness  Don’t know |  |
| 15.  16. | How can anemia be prevented/treated?  **Tick all that apply** | Taking recommended iron and folic acid supplement  Eat a diet rich in iron and folic acid  Taking Vitamin A and B12 supplements  Taking in a lot of coffee, tea, and sugar  prevent malaria by sleeping under mosquito nets  Don’t know |  |
|  | What are some of the consequences of anemia? **Tick all that apply** | Diarrhea  Reduced attention span  Poor learning performance  Speedy recovery from diseases  Low resistance to infection  Decreased physical activity  Don’t know |  |
| **Knowledge on Weekly iron folic acid supplementation (WIFAS)** | | | |
| 17. | Do you know about the weekly iron-folic acid supplements? | Yes  No |  |
| 18 | Why to give the iron and folic acid tablet every week? | To prevent anemia in adolescent girls  To stop diarrhea in girls  Don’t know |  |
| 19 | Why do you think girls are given iron and folic tablets rather than boys? **Tick all that apply** | Girls are at risk of anemia  Boys are healthier than girls  Girls lose blood every month  Don’t know |  |
| 20 | What are the benefits of WIFAS? | Improve concentration and performance in school  Increased blood volume  Prevent malaria  It prevents iron and folic acid deficiency  Improve our general health  Prevent diarrhea  Don’t know |  |
| 21 | What are some of the food items that inhibit/decrease iron absorption? **Tick all that apply** | Coffee  Vegetables  Tea  Milk and milk products  Fruits  Don’t know |  |
| 22 | Which of the following are side effects of taking iron and folic acid tablet? **Tick all that apply** | Stomach ache  Vomiting  Black stool  Constipation  Diarrhea  Severe pains  Don’t know |  |

| **SECTION C**  Please describe the foods (meals and snacks) that you ate yesterday during the day and night, whether at home or outside the home. Start with the first food eaten in the morning | | | | |
| --- | --- | --- | --- | --- |
| **Sn** | **Food Groups** | **Food categories** | **Food items** | **Consumed within last 24 hours** |
| C001 | Grains, white roots and tubers and plantains | Foods made from grains | Maize, rice, barley, millet, sorghum, wheat | Yes …1 No…..2 |
|  |  | white roots and tubers and plantains | Potatoes, turnip | Yes….1 No......2 |
| C002 | Pulses (beans, peas and lentils) | Pulses (beans, peas and lentils) | Dal, pulses, pea, beans, soyabean, chana Dal | Yes …1 No…..2 |
| C003 | Nuts and seeds | Nuts and seeds | Almond, chestnut, walnut, peanuts | Yes….1 No…..2 |
| C004 | Dairy | Milk and milk products | Milk, yoghurt, cheese | Yes …1 No…..2 |
| C005 | Meat, poultry and fish | Organ Meat | Liver, heart, blood products, kidney, gizzard | Yes …1 No…..2 |
|  |  | Meat and poultry | Beef, goat, lamb, mutton, pork, buffalo, rabbit, chicken, duck | Yes….1 No…..2 |
|  |  | Fish | Fish | Yes …1 No…..2 |
| C006 | Eggs | Eggs | Chicken eggs, duck eggs | Yes …1 No…..2 |
| C007 | Dark green leafy vegetables | Dark green leafy vegetables | Spinach, pumpkin leaves, amaranths, mustard leaves, broccoli, carrot leaves | Yes ...1 No….2 |
| C008 | Other Vit. A rich fruits and vegetables | Vitamin A-rich vegetables, roots and tubers | Carrot, pumpkin, sweet potato | Yes ...1 No….2 |
|  |  | Vitamin A-rich fruits | Mango, papaya | Yes …1 No…..2 |
| C009 | Other vegetables | Other vegetables | Cabbage, cauliflower, corn, tomato, bamboo shoots, mushroom, cucumbers | Yes ....1 No…..2 |
| C010 | Other fruits | Other fruits | Apple, banana, grapes, guava, amla, lemon, lime, orange, pear, pineapple, strawberry, watermelon | Yes ...1 No….2 |

| **SECTION D: Biological test** | | |
| --- | --- | --- |
| **Hemoglobin level (g/dl)** | **...........................** |  |
