# Supplementary material for: Prevalence of anemia and its associated factors among adolescent girls on Weekly Iron Folic Acid supplementation (WIFAS) implemented and non-implemented schools at Tokha municipality, Kathmandu
Source: PLOS Glob Public Health. 2024 Jan 26;4(1):e0002515. doi: 10.1371/journal.pgph.0002515 (PMC10817172; doi:10.1371/journal.pgph.0002515)
Supplement: Attachment — Submitted filename: Response to Reviewers.docx [file pgph.0002515.s003.docx]

**Rebuttal letter**

Dear editor,

We are grateful for your time and effort for reviewing our manuscript (PGPH-D-23-01329). Thank you for your insightful comments and constructive feedback. We have revised our manuscript according to your and the reviewers’ comments.

| **Comments by Editor** | | **Comments addressed** | |
| --- | --- | --- | --- |
| 1 | **Study setting, design, and population** The description of the study settings gives helpful background for the readers. However, it would be helpful to have more detailed information, such as the number of schools that have selected for the study in WIFAS implemented and not implemented of Tokha municipality. | 1 | Eight WIFAS and eight non-WIFAS implemented schools were selected for the study.  **It has been well explained in the line number 82 to 94, page no. 4.** |
| 2 | **Sample size calculation and sampling technique** It's a worthy you highlighted the method used to calculate sample size and the reference to a comparable research for explanation. However, it is better to provide more details information about the parameters assumed for this study. Furthermore, it would be worthy to describe on what basis a design effect of 1.5 was decided. Authors mentioned the use of Probability Proportional to Size (PPS) technique for cluster selection. This seems confusion to the readers, therefore it would be helpful to provide a brief explanation of what PPS is and how it was applied in for study. |  | The sample size was calculated using the formula of difference between two proportion.  n=(Z_(1-α/2) +_ Z_(1-β)_)²_*_ p_1_ (1-p_1_)+p_2_ (1-p_2_)/( p_1-_p_2_)²  where,  proportion of outcome from WIFAS non implemented school (p_1_) = 64.4%=0.644  proportion of outcome from WIFAS implemented school (p_2_) = 51%=0.51  power (1-β) =80%=0.8  Z_alpha_ value=1.96 at 95 % Confidence interval  Z_beta_ value=0.84 at corresponding 80% power  With design effect of 1.5 and assuming a non-response rate of 10%, the final sample of 602 was calculated.  (301 sample from each WIFAS implemented and non-implemented schools)  (Source: (Hameed S et.al, 2022)  **(Please refer to page number 4, line number 96)**  Design effect 1.5 was selected in reference to a national survey, Nepal Demographic Health Survey, 2022. (**Please refer to line number 109, page number 5)**  For detail explanation on PPS, **please refer to the line number 119, page no.5)** |
| 3 | **Discussion** Contextualize the Study: It would be constructive to begin discussions by providing a quick summary of the importance of addressing anemia among adolescent of Nepal. It may include information on the health and economic effects of anemia, as well as why this study is important, and then emphasizing WIFAS's success in terms of elements of WIFAS that contribute to its effectiveness, and how does it compare to other interventions as you demonstrated the effectiveness of WIFAS in reducing anemia |  | It has been described in the first paragraph of the discussion section in **line number 301**  The health and economic effects of anemia including its importance have been addressed in **line number 304**  WIFAS effectiveness in reducing the prevalence of anemia has been described **in line number 321 to 327** |
| 4 | **Conclusion** These findings have strong policy implications for enhancing adolescent girl’s health. As a result, it is recommended that you discuss policy implications based on your results. |  | The conclusion of the study has been drawn on the basis of the findings after calculating the adjusted odds ratio.  (**For detail please refer to page number 21, paragraph 1^st^)**  **Implications of the findings**  The prevalence of anemia was found lower among WIFAS implemented schools where there was the provision of a school heath nurse. Therefore, a school health nurse has to be recruited in every government and non-government school to motivate and counsel adolescent girls on the benefits and consumption of WIFAS.  The findings of the study can be utilized for the evidence-based public health policy formulation addressing WIFAS non-implemented schools, community-based schools and out of school adolescent girls, where they are at risk of anemia.  Effective communication and implementation of WIFAS program can lead to improved adolescent health and ultimately to societal health and wellbeing.  **(Please refer to line no. 426 to 434, page no. 22)** |

**Rebuttal letter**

Dear reviewer 1,

We would like to appreciate your time and effort in going through the manuscript and provide comments. Thank you for your generous comments on the manuscript and we have hereby tried to address the comments.

| **Comments by reviewer -1** | | **Comments addressed** | |
| --- | --- | --- | --- |
| 1 | Grammatical issues should have been revised in the whole manuscript. | 1 | Thank you for your comments. I have addressed grammatical issues in the manuscript in the following line number.  (**45,77,87,91,98,112, 132,149,165,191,226,297,433)** |
| 2 | Providing some more global statistics on anemia would make the introduction better. Moreover, condition of anemia in Nepal should have been included. | 2 | I have incorporated global anemia data from WHO, 2022 and national data on anemia from a national survey, Nepal Demographic Health Survey, 2022  **Please refer to line number (54 to 59)** |
| 3 | Mentioning the exact equation to calculate the sample size would be better. | 3 | The sample size was calculated using the formula of difference between two proportion.  n=(Z_(1-α/2) +_ Z_(1-β)_)²_*_ p_1_ (1-p_1_)+p_2_ (1-p_2_)/( p_1-_p_2_)²  **Please refer to line number (99)** |
| 4 | In the prevalence section of the results, frequency of the participants should have been omitted and more focus should have been given to the percentage | 4 | In the prevalence section of the results, the frequency has been omitted and the percentage has been mentioned.  **Please refer to line number ( 230,231)** |
| 5 | Results should have been described in past tense | 5 | This comment has been addressed throughout the manuscript. |
| 6 | There is an inconsistency in the use of “non-implemented schools”. Author should correct all the “not implemented schools” to “non-implemented schools”. | 6 | All not implemented schools were corrected to  non-implemented schools throughout the manuscript. |
| 7 | Authors have mentioned both “multivariable logistic regression analysis” and “multivariable logistic regression analysis”. Author should clarify about which one he/she used; As there is a difference between these 2 (two) analysis method | 7 | Multivariate analysis was changed to multivariable logistic regression analysis  **Please refer to line number (281)** |
| 8 | Limitation of the study” should have been mentioned in a separate section. | 8 | Limitations of the study have been mentioned in a separate section.  **Please refer to line number (290),page number 16**. |
| 9 | “In the study, the impact of deworming on anemia prevalence couldn’t be assessed. The level of serum ferritin couldn’t be assessed to measure iron storage”- This part of page 5 should have been included as the limitation of the study. | 9 | Yes, this part has been included in the section of “Limitation of the study”. |
| 10 | The knowledge of anemia and the knowledge of WIFAS was calculated by adding all relevant 19 and 15 knowledge items questions respectively. A correct answer for each item was scored as “1” and incorrect answer was scored as “0”- This part of page 10 should have been included in the method section of the study. | 10 | Yes, this part has been included in the method section of the study.  **Please refer to line number (159)** |
| 11 | “Analysis of association of socio-demographic, knowledge on anemia and WIFAS and dietary diversity with anemia”- This headline should be reworded as- “Association of socio-demographic characteristics, knowledge of anemia and WIFAS and dietary diversity with anemia” | 11 | Yes, the heading has been reworded as suggested, “Association of socio-demographic characteristics, knowledge of anemia and WIFAS and dietary diversity with anemia”  **Please refer to line number (251)** |

Dear reviewer 2,

We would like to appreciate your time and effort to go through the manuscript and provide comments. Thank you for your generous comments on the manuscript and we have hereby tried to address the comments.

| **Comments by reviewer -2** | | **Comments addressed** | |
| --- | --- | --- | --- |
| 1 | The data collection procedures adopted lacks details about how the questionnaires were distributed, collected, and managed. Information on data collection supervision, training of data collectors, and quality control measures would enhance the methodology's robustness. For instance, how were the students approached, informed about the study, and how was their consent obtained? | 1 | This comment has been well explained in **page** **number 6**, data collection tools and procedures and under ethical approval and consent section, p**age number 9.**  **Please refer to line number ( 129 to 137 ) and (190 to 199)** |
| 2 | The knowledge on anemia, knowledge on WIFAS, dietary diversity and compliance-related questionnaire was adapted from different published articles. Which articles are these and what exactly did the authors adopt from each of the articles. | 2 | A standard questionnaire from the Adolescent Nutrition Health Survey 2014; Journal of nutrition and metabolism by SD Dubik et.al 2019 and dietary diversity questionnaire from FFQ, 2016 FAO were adopted. |
| 3 | More information about the process of pretesting, findings from pretesting, and the nature of modifications made to the questionnaire after pretesting and how they improved the tool should be explained. | 3 | The pretesting of the tool was done in 10% of sample size (n=60) in one WIFAS-implemented, and one non-implemented school in nearby Tarkeshwor municipality, Kathmandu. No modification on the questionnaire was made. Only the flow and sequence of the questions were changed.  On the basis of the findings of pretesting, respondents were provided brief content orientation on anemia, WIFAS and questions skipping patterns to ensure data quality.  **(For details, please refer page no. 6 )** |
| 4 | Did the questionnaire undergo any content validation or reliability testing? if yes explain the process and the outcome. | 4 | An already validated questionnaire was used. So, no content validation was performed.  For reliability testing, pre-testing was done among 10% of the sample size. |
| 5 | The authors should include the duration of the data collection period. | 5 | The data was collected after receiving ethical approval from IRC from December 18, 2022 to January 25, 2023. |
| 6 | The section could provide a brief outline of the types of the specific statistical analyses conducted and the purpose of each. | 6 | Descriptive statistics (including means, standard deviations, frequencies and percentage) was calculated  Purpose  To summarize or describe the data  A Chi-square test  Purpose  To determine the relationship between the independent variables and with outcome variable (anemia)  The test of multi-collinearity  Purpose  To determine the relationship or interdependence between independent variables themselves  P-value  Purpose  To determine the statistical significance of less than 0.05 at 95% confidence interval.  Multivariable logistic regression analysis  Purpose  To determine the independent association between anemia (dependent variable) and explanatory variables (sociodemographic, Knowledge related variables and dietary diversity)  Adjusted odds ratio  Purpose  To determine the strength of association, the odds of occurring outcome event (anemia) after adjusting other confounding variables.  **(Please refer to page no 9, data analysis)** |
| 7 | The section mentions some limitations, but it could be more comprehensive. Addressing limitations associated with the study design, data collection, and analysis adds to the study's credibility. | 7 | The limitations associated with the study design, data collection, and analysis have been well explained under the title of limitation.  **Please refer to page number ( 16)** |
| 8 | While the non-response rate assumption of 10% is mentioned, it could be helpful to provide reasons for this assumption and acknowledge potential implications. | 8 | With the assumption of things like non-consent, non-participation and incomplete response among participants 10% non-response rate was chosen. Its potential implications were that if the assumed non-response rate deviated from 10%, it could impact on generalizability of study findings and lead to bias in the results. So, 10% non-response rate was taken. |
| 9 | The authors mentioned obtaining consent from parents before the blood draw, but it doesn't elaborate on how parental consent was obtained. Clarifying this process would strengthen the ethical considerations. | 9 | Parental consent was taken one day before data collection and blood was withdrawn from only those whose parental consent as well as student assent forms were obtained.  The parental consent and assent forms were attached in the supplementary section herewith.  **(For details please refer to line number 129 to 137,146 to 158, under data collection tools and procedures,190 to 204 under ethical issues )** |
| 10 | What were the steps taken to ensure data quality, such as data validation checks or inter-rater reliability checks for the self-administered questionnaire? | 10 | To ensure data quality, an already validated tool was used.  Q-Q plot displayed the data set was normally distributed.  Pre-testing was done to ensure the reliability of the tool.  The completeness, consistency and timeliness were assured by research supervisors and nutrition experts.  WHO recommended HemoCue Hb 301 system was used for accuracy of the data.  At first, the researcher and lab technician themselves measured their Hb level using the system to ensure the accuracy of the device.  **(please refer page number 6)** |
| 11 | What was the response rate? | 11 | All the respondents participated in the study. They were excited to know their hemoglobin level. It was a 100% response rate. |
| 12 | What are the implications of your findings? | 12 | The prevalence of anemia was found lower among WIFAS implemented schools where there was provision of school heath nurse. Therefore, a school heath nurse has to be recruited in every government and non-government schools to motivate and counsel adolescent girls on the benefits and consumption of WIFAS.  The findings of the study can be utilized for the evidence based public health policy formulation addressing WIFAS non-implemented schools, community based schools and out of school adolescent girls, where they are at risk to anemia.  Effective communication and implementation of WIFAS program can lead to improved adolescent health and ultimately to societal health and wellbeing.  **Please refer to page number (22)** |

Dear reviewer 3,

We would like to appreciate your time and effort to go through the manuscript and provide comments. Thank you for your generous comments on the manuscript and we have hereby tried to address the comments.

| **Comments by reviewer -3** | | **Comments addressed** | |
| --- | --- | --- | --- |
| **Major issues** | |  | |
| **Methods** | | | |
| 1 | I don’t understand your sample size calculation section (middle of page 7 of the pdf); I would expect that you need to include an assumed prevalence of anemia as well as an assumed effect size of WIFAS to conduct the power calculation. | 1 | The sample size was calculated using the difference between two proportion formula.  n=(Z_(1-α/2) +_ Z_(1-β)_)²_*_ p_1_ (1-p_1_)+p_2_ (1-p_2_)/( p_1-_p_2_)²  where,  proportion of outcome from WIFAS non implemented school (p_1_) = 64.4%=0.644  proportion of outcome from WIFAS implemented school (p_2_) = 51%=0.51  power (1-β) =80%=0.8  Z_alpha_ value=1.96 at 95 % Confidence interval  Z_beta_ value=0.84 at corresponding 80% power  With design effect of 1.5 and assuming a non-response rate of 10%, the final sample of 602 was calculated.  (301 sample from each WIFAS implemented and non-implemented schools)  Source: (Hameed S et.al, 2022)  **(please refer to page number 4 and 5, in line number 96)** |
| 2 | Furthermore, it seems essential to include additional detail on how the eight schools were selected from the WIFAS and non-WIFAS groups. If they were selected uniformly at random, then your results are more likely to be generalizable than if they were selected in some non-random manner. | 2 | There were altogether eight WIFAS implemented government schools in Tokha municipality and all were taken for study. Whereas eight WIFAS non-implemented schools were selected randomly on the basis of similar socioeconomic characteristics of respondents from the same ward of WIFAS implemented schools to ensure comparability. This part has been rewritten in the manuscript.**(please refer to line number 88 to 94)** |
| **Results** | | | |
| 1 | I would like a report on the participation rate and amount of missing data. Please also describe how you handled missing data in your methods section. | 1 | After a careful understanding of the study design, methodology, purpose, potential risks and benefits and the right of respondents to withdraw at any time, all the participants participated in the study.  It was a 100% response rate. |
| 2 | I would like all of your Table 2 characteristics reported for the WIFAS and non-WIFAS schools separately, to see if these schools seem similar on these dimensions. | 2 | This comment has been rigorously addressed regarding the sociodemographic characteristics of WIFAS and non-WIFAS implemented schools separately.  **(Please refer to page no. 11 table 2)** |
| **Supporting information** | | | |
| 1 | I think you should include the individual participant data (appropriately anonymized) as an additional supporting document | 1 | Personal identity information was not included in the questionnaires and to maintain the anonymity of respondents, unique codes were used during the entire period of study. |
| **Minor issues** | | | |
| 1 | Page 5, line 3: “the fond ages of ten to nineteen”, the word fond sound too informal to me | 1 | The term fond has been omitted.  **(please refer to line number 45)** |
| 2 | Page 5, line 3: “this is a remarkable stage of life”, this phrase sounds awkward to me | 2 | The term remarkable has been omitted.  **(please refer to line number 45/46)** |
| 3 | Page 5, line 7: reference 3 seems overly focused, with a title about girls in Central Kerela. Please include a more general reference here instead. | 3 | This reference of Central Kerela has been replaced by another more general reference  (Hemoglobin concentrations for the diagnosis of anemia and assessment of severity. Vitamin and Mineral Nutrition Information System. Geneva, World Health Organization, 2011 (WHO/NMH/NHD/MNM/11.1) **(please refer to reference number 3, line no.454)** |
| 4 | Page 5, line 10: double check if these references are what you intended; ref [4] seems like it might be intended for elsewhere. | 4 | This reference has been checked and corrected. |
| 5 | Page 5, line 9 from bottom: is there an evidence synthesis that you can cite here, such as a systematic review and meta-analysis? | 5 | Yes, a systematic review and meta-analysis was conducted in Bangladesh from 1997 to 2019.  **(please refer to line number 70 to 73)** |
| 6 | Page 5, final paragraph: this has a lot in common with the previous paragraph, see if you can merge them | 6 | Yes, the paragraph was merged. |
| 7 | Page 5, line 3 from bottom: as an non-expert reader, I would like to know more about the state of WIFAS and its context. Has this been piloted extensively before? Has it been used programmatically? | 7 | WIFAS is a nationwide program.  The following table gives a brief overview of the program. |
| Supplement composition | | | Iron: 60 mg of elemental iron Folic acid: 400 μg |
| Number | | | One supplement per week |
| Duration and time interval between periods of supplementation | | | 3 months of supplementation (13 weeks) followed by 3 months (13 weeks) of no supplementation after which the provision of supplements should restart(13 weeks). |
| Target group | | | All adolescent girls (10-19) |
| Total duration of supplementation | | | 26 weeks per year |
| 8 | Page 5, line 2 from bottom: “an interval of three months” is not clear to me, can you rephrase? | 8 | It means WIFAS is provided for three months, stopped for next three months and again provided for another three months.  Only provided for six months.(As aforementioned in table) |
| 9 | Page 5, last line: ref 14 is almost 500 pages long, so please include the page of the report with this information | 9 | Yes, page number has been included.  (i.e pg no. 100)  **(Please refer to reference in line number 487, reference no. 15)** |
| 10 | Page 6, line 8-9: phrasing of “the study methods was quantitative method through…” is an awkward sentence, see if you can rephrase it. | 10 | The sentence has been rephrased.  **(Please refer to line number 87)** |
| 11 | Page 7, paragraph 1: how many schools did you sample, how many students total? What was the response rate? | 11 | Eight WIFAS implemented and eight WIFAS non implemented schools (sixteen schools in total were sampled)  100%response rate.  There were 1234 girl students in WIFAS implemented and 483 girl students from WIFAS non implemented schools from grade six to ten only.  **(Please refer to page number 4, line no. 89 to 94 )** |
| 12 | Page 7, last line: “and lab technician was used for …” I find this phrasing awkward | 12 | It has been rephrased.  **(please refer to line number 132 )** |
| 13 | Page 8, line 6: does the HemoCue Hb30 require a blood sample? More information on this for the non-expert reader would be helpful. | 13 | HemoCue Hb301 system is an effective tool that requires few drops of blood from participant for hemoglobin estimation as it is diagnostically accurate and recommended by WHO to diagnose anemia. To draw blood from the finger, a sterile lancet was used. Blood was collected on a microcuvette after setting to zero and then inserted into the HemoCue device.  **(Please refer to line number 134 to 136,141 to 143 )** |
| 14 | Page 9, table cell on Knowledge on anemia and WIFAS: what was the median, and what did you do with scores with value exactly equal to it? | 14 | The median mark obtained on knowledge of anemia was six and for knowledge on WIFAS was five.  The knowledge on anemia and WIFAS was considered good if the score was exactly equal to median and above.  (please refer to line no.163) |
| 15 | Page 10, line 4-5 from bottom: is the season during which the data was collected relevant? | 15 | Yes the season was relevant, it was collected before the start of winter vacation of the schools. |
| 16 | Page 11, table 2: is ethnicity/caste relevant to anemia deficiency in this population? I would be interested to know more about this in the methods and discussion. | 16 | There was no significant association between ethnicity and anemia in the study. |
| 17 | Page 12, Table 2 continued: there might be more rows than necessary in this table, consider if you can simplify the rows by grouping some categories or leaving things out. | 17 | Yes, the rows were categorized.  **(please refer to table 2, page number 11 )** |
| 18 | Page 13, line 3-4: your observation that there is no severe anemia in non-WIFAS schools made me wonder again if there is some systematic bias being introduced by the selection of which schools to implement a WIFAS program | 18 | In the study, non-WIFAS were private schools. There was no severe anemia in non-WIFAS schools, it might be due to adequate dietary diversity. They had consumed at least five or more food groups. |
| 19 | Page 13, paragraph 2: I would like to see a comparison of distributions here, since the means are so similar but the percent anemic have this important difference. A plot overlaying the histogram of hemoglobin levels in the WIFAS and non-WIFAS schools could do this, or a quantile-quantile plot comparing these distributions even more directly might be appropriate. | 19 | A dataset and Q-Q plot indicated that the data was roughly normally distributed because the nearly straight line closely followed the diagonal line.  **(Please refer to line number 240, pg no. 12)** |
| 20 | Page 13, paragraph 3: the approach to the knowledge questions should be described in the methods section, and when you move it there also double check the values, because it seems like it might be inconsistent, e.g. 19 and 15 items add up to maximum of 12 and 10. | 20 | Knowledge related questions have been moved to method sections and median of the score was calculated on the basis of the score obtained by each participant.  **(Please refer to page no7, last paragraph)** |
| 21 | Page 13, paragraph 4: This is unclear, and appears potentially inconsistent. 59.1% is not two times more than 54.2, for example, so I’m probably misunderstanding your claims. 78.4 is not two time more than 46.5, either. | 21 | **This unclear issue has been addressed on page no. 207 to 211** |
| 22 | Page 13, paragraph 5: this phrasing is a bit confusing and awkward as well. | 22 | This confusing phrasing has been removed. |
| 23 | Page 13-14: I think I need to know about the functional form of your regression model in the methods section to appreciate the implications of this. Is this a sequence of univariate regression models or a single multivariate regression. | 23 | Multivariable logistic regression analysis was performed after multi-collinearity testing. Outcome variable were categorized into anemic and non-anemic.  The functional form of regression model:  y= log(p/(1-p))= β0 + β1*x1 + … + βk*xk = Bt. X , for specific values of x |
| 24 | Page 14, paragraph 2: I would appreciate a table of figure showing these coefficients **Variables in the Equation** \| \| \| \| \| \| \| \| \|; \| \| B \| S.E. \| df \| Sig. \| **Exp(B)** \| 95% C.I.for EXP(B) \| \|; \| \| \| \| \| \| \| Lower \| Upper \|; Step 1^a^ \| Category of Age \| -.204 \| .390 \| 1 \| .600 \| **.815** \| .380 \| 1.750 \|; \| classes \| .763 \| .391 \| 1 \| .051 \| **2.145** \| .997 \| 4.616 \|; \| father education \| 1.317 \| .355 \| 1 \| **.000** \| **3.733** \| 1.862 \| 7.485 \|; \| Mother occupation \| -.060 \| .453 \| 1 \| .895 \| **.942** \| .388 \| 2.290 \|; \| Mother education \| .331 \| .406 \| 1 \| .414 \| **1.393** \| .629 \| 3.085 \|; \| Knowledge on Anemia Category(1) \| -.417 \| .271 \| 1 \| .124 \| **.659** \| .388 \| 1.121 \|; \| WIFAS Knowledge Category(1) \| -.229 \| .294 \| 1 \| .436 \| **.796** \| .448 \| 1.415 \|; \| Food Diversity Level(1) \| -2.490 \| .279 \| 1 \| **.000** \| **.083** \| .048 \| .143 \|; \| Type of School(1) \| -1.139 \| .306 \| 1 \| **.000** \| **.320** \| .176 \| .583 \|; \| Constant \| 2.560 \| .516 \| 1 \| .000 \| **12.942** \| \| \| | | |
| 25 | Page 14, last line: mention table 3 earlier. | 25 | Yes it has been corrected as mentioned in **line number 276** |
| 26 | Page 17, last line of table: the odds ratio for inadequate dietary diversity seems important, and could perhaps be a larger part of the results and discussion. Was this something that you would have expected a priori? If it is a surprise, that is all the more reason to call attention to it. ???? | 26 | In my study, non-WIFAS implemented were (private schools) that had better access to food diversity than WIFAS implemented (government) schools, but anemia prevalence was higher due to the lack of IFA supplementation. Government schools have comparatively lower access to food diversity, but anemia prevalence was found to be lower due to the fact that WIFAS was implemented. |
| 27 | Page 18, line 2: you say that this is low cost, but you need to present some costing evidence in the discussion (and describe how you obtained it in the methods) to make this claim in the discussion. | 27 | It has been mentioned in first paragraph under discussion section.  **(please refer to page number 17, line number 3 to 5)** |
| 28 | Page 18, line 5: the term “WIFAS not implemented schools” has always been awkward in this paper, but this time particularly confused me. I suggest you use something like “non-WIFAS”. | 28 | WIFAS schools and non-WIFAS schools have been used throughout the paper. |
| 29 | Page 18, line 7: I don’t think it is appropriate to call this statistically significant without making the argument that it can be treated like a random sample of schools which have been randomly assigned to have WIFAS or not. | 29 | Thank you for the critique. Therefore, only significant has been used. |
| 30 | Page 18, line 6 from bottom: I would be very interested in a visual representation of the effectiveness of WIFAS from different studies and how the results of this study fit into the existing evidence base. | 30 | 1. The overall findings indicate that WIFAS intervention reduced the prevalence of anemia among adolescent girls from 79.5% to 58%, with a mean increase in hemoglobin of 1.3 g/dl among girls **(Please refer to reference number 10).** 2. The prevalence of anemia was 38% at the start of the study and had dropped to 26% by the end of the one-year intervention. The average hemoglobin concentration rose by 0.37gm/dl **(Please refer to reference number 11).** 3. IFA consumption was positively associated with Hb and negatively associated with anemia, with the prevalence of anemia decreasing from 25.1% to 19.6% during the first school year of the WIFAS intervention **(Please refer to reference number 12).** 4. Anemia was prevalent among schoolchildren in 51% of government schools (WIFAS implemented) and 64.4% of private schools (non-WIFAS implemented). In government and private school girls, the mean hemoglobin was 11.77 1.41 and 11.34 1.49, respectively **(Please refer to reference number 16).** |
| 31 | Page 19, line 1-3: your comment about poor compliance made me wonder if your analysis is among people who were treated or among everyone in the school that is offering the treatment? | 31 | Sorry to make you confused.  My analysis is among everyone in the school.(compliance has been omitted) |
| 32 | Page 19, line 4-7: which paper are you referring to here? | 32 | It has been referenced new. **(Please refer to reference number 10-13)**. |
| 33 | Page 19, line 10: mentioning that a different study ran for 6 months made me wonder how long was this WIFAS program running before your study was conducted? | 33 | I mean that if intervened for 6 months how much would hemoglobin increase? In my study area program was launched for more than 18 months |
| 34 | Page 19, line 10 from bottom: “This would result adolescent comply” is awkward phrasing. What was the compliance rate in the WIFAS schools? | 34 | Compliance is not a variable of interest in my study. So, it has been omitted from manuscript. |
| 35 | Page 19, final paragraph: there is some awkward phrasing in the second sentence here. | 35 | This awkward phrase has been removed. |
| 36 | Page 20, last line: 12.1 times *more likely*? Or 12.1 times *odds ratio* to be anemic? | 36 | It is 12.1 times the odds ratio. |
| 37 | Page 21, paragraph 2: some of this should go in the methods section. | 37 | **Yes, it has been moved in method section.**  **(Please refer to page number 4)** |
| 38 | Page 22, line 2-3: the conclusion that WIFAS significantly reduces anemia strikes me as too bold without more justification for how you have avoided potential bias from which schools implemented WIFAS and which schools were sampled. | 38 | The conclusion of the study has been drawn on the basis of the data findings after calculating the adjusted odds ratio. First paragraph of conclusion, **page number 21, line number 405 to 416)** |
| 39 | Page 22, line 5: odds reported in results section were 30% higher, not 300% higher! | 39 | Yes, it is 30% higher. |
